# 2-Hydroxymelatonin, Rather Than Melatonin, Is Responsible for RBOH-Dependent Reactive Oxygen Species Production Leading to Premature Senescence in Plants

**DOI:** 10.3390/antiox10111728

**Published:** 2021-10-29

**Authors:** Hyoung Yool Lee, Kyoungwhan Back

**Affiliations:** Department of Biotechnology, College of Agriculture and Life Sciences, Chonnam National University, Gwangju 61186, Korea; xanthine@naver.com

**Keywords:** abscisic acid, ethylene, 2-hydroxymelatonin, mitogen-activated protein kinase, melatonin, RBOH, ROS, senescence

## Abstract

Unlike animals, plants amply convert melatonin into 2-hydroxymelatonin (2-OHM) and cyclic 3-hydroxymelatonin (3-OHM) through the action of melatonin 2-hydroxylase (M2H) and melatonin 3-hydroxylase (M3H), respectively. Thus, the effects of exogenous melatonin treatment in plants may be caused by melatonin, 2-OHM, or 3-OHM, or some combination of these compounds. Indeed, studies of melatonin’s effects on reactive oxygen species (ROS) production have reported conflicting results. In this study, we demonstrated that 2-OHM treatment induced ROS production, whereas melatonin did not. ROS production from 2-OHM treatment occurred in old arabidopsis leaves in darkness, consistent with an ethylene-mediated senescence mechanism. Transgenic tobacco plants containing overexpressed rice *M2H* exhibited dwarfism and leaf necrosis of the upper leaves and early senescence of the lower leaves. We also demonstrated that 2-OHM-mediated ROS production is respiratory burst NADPH oxidase (RBOH)-dependent and that 2-OHM-induced senescence genes require ethylene and the abscisic acid (ABA) signaling pathway in arabidopsis. In contrast to melatonin, 2-OHM treatment induced senescence symptoms such as leaf chlorosis and increased ion leakage in arabidopsis. Senescence induction is known to begin with decreased levels of proteins involved in chloroplast maintenance, including Lhcb1 and ClpR1. Together, these results show that 2-OHM acts as a senescence-inducing factor by inducing ROS production in plants.

## 1. Introduction

In plants, melatonin is a multifunctional molecule that displays a diverse set of physiological functions in plant growth and development ranging from seed germination to seed longevity and post-harvest preservation [1,2,3,4]. Melatonin also confers great ecological benefits when plants are challenged with adverse conditions, including diverse biotic and abiotic stresses [5,6]. The mechanisms by which melatonin plays these physiological roles are closely dependent on its intrinsic antioxidant activity and its function as a signaling molecule in association with its receptor and downstream signaling cascades [7,8].

In both animals and plants, melatonin is synthesized from tryptophan in a process requiring four enzymes [9]. The last two steps of this process are well conserved in all organisms, comprising serotonin *N*-acetyltransferase (SNAT) and *N*-acetylserotonin *O*-methyltransferase (ASMT), in biosynthesis order. Under certain conditions, this order is reversed by producing 5-methoxytryptamine through ASMT, followed by melatonin synthesis through SNAT [10]. In contrast to conserved melatonin biosynthesis, melatonin catabolism differs greatly between animals and plants, mainly in that it is almost an end product in animals, but a precursor for further metabolites in plants. These melatonin metabolites are 2-hydroxymelatonin (2-OHM) and cyclic 3-hydroxymelatonin (3-OHM), although both are also produced nonenzymatically in animals as degradation products [11]. In contrast, both 2-OHM and 3-OHM are predominantly and enzymatically produced in plants. Thus, these metabolites may possess their own functions in plants. In support of this hypothesis, 2-OHM is produced at a rate 300-fold higher than that of melatonin in plants [12], and exogenously treated melatonin is rapidly converted into 2-OHM and 3-OHM by melatonin 2-hydroxylase (M2H) and melatonin 3-hydroxylase (M3H), respectively, in rice seedlings [13]. The rapid and efficient conversion of melatonin into either 2-OHM or 3-OHM raises the question of whether the functions or phenotypes of plants treated with exogenous melatonin result from either melatonin, 2-OHM, or 3-OHM alone, or some combination of these compounds.

Previous studies have produced inconsistent reports on the effects of melatonin treatment, especially on reactive oxygen species (ROS) production in plants. Because melatonin is an antioxidant signaling molecule, its treatment does not alter ROS levels in healthy control plants and can significantly decrease ROS levels in plants challenged with many stresses [14], as has been shown in wheat [15] and cucumber [16] stressed with heavy metals. In marked contrast, exogenous melatonin treatment increases ROS levels in healthy control plants but significantly decreases ROS under stress conditions such as cold [17] or salt [18]. However, another study found that melatonin treatment did not alter ROS levels in control plants but induced more ROS than the control under salinity stress [19]. Surprisingly, endogenous melatonin synthesis was recently reported to closely parallel ROS production in arabidopsis, where it modulates diurnal stomatal closure [20]. ROS production in response to melatonin treatment is regulated by respiratory burst NADPH oxidases (RBOHs) [19,20,21,22]. Together, these findings indicate that melatonin acts as both an antioxidant and a pro-oxidant in plants, as it does in animals [23].

Based on this dual property of melatonin as antioxidant and pro-oxidant, and the presence of predominant melatonin metabolites such as 2-OHM in plants, we hypothesized that 2-OHM may be responsible for ROS synthesis when healthy plants are challenged with melatonin. This hypothesis is also supported by the findings of a previous study, in which an attempt to overexpress M2H, a major enzyme for 2-OHM synthesis, failed to generate transgenic rice plants due to the necrosis of embryogenic rice calli during the regeneration process [24].

In this study, we examined ROS production in plants treated with either melatonin or 2-OHM in arabidopsis to determine which is responsible for plant functions and phenotypes associated with transgenic tobacco (*Nicotiana benthamiana*) overexpressing the rice *M2H* gene. We also explored the roles of ethylene and the abscisic acid (ABA) pathways in 2-OHM-induced ROS production in arabidopsis.

## 2. Materials and Methods

### 2.1. Transgenic Tobacco Plants Overexpressing Rice M2H

Full-length rice *M2H* cDNA (AK119413) was amplified by polymerase chain reaction (PCR) using a primer set (forward primer: 5′-GGG GAC AAG TTT GTA CAA AAA AGC AGG CTC CAT GCC CGC CGT GGC CGG G-3′; reverse primer: 5′-GGG GAC CAC TTT GTA CAA GAA AGC TGG GTT CAG GGT TTG TCG AT-3′), gel-purified, and cloned into the pDONR221 Gateway vector (Invitrogen, Carlsbad, CA, USA) via BP recombination. The resulting pDONR221:M2H entry vector was then recombined with the pK2GW7 Gate destination vector [25] via LR recombination to form pK2GW7-M2H, which was transformed into *Agrobacterium tumefaciens* GV2260. Tobacco transformation was conducted according to Duan et al. [26]. T_1_ seeds were screened on Murashige and Skoog (MS) medium containing 200 mg/L kanamycin. After selfing the T_1_ plants, T_2_ homozygous tobacco lines were selected and used in this experiment.

### 2.2. Plant Material and Growth Conditions

*Arabidopsis thaliana* ecotype Columbia (Col-0) plants were grown in a growth room at 23 °C with 50% relative humidity under 12-h light/12-h dark conditions (50 μmol m^−2^ s^−1^) as described previously [27]. Fluorescent lights (Osram, Seoul, Korea), half at 6500 K (865 FPL36EX-D) and half at 4000 K (840 FPL36EX-W) were used as the light sources for the arabidopsis room. Tobacco (*N. benthamiana*) plants were grown in soil under long-day growth conditions (16-h light/8-h dark) at 28 °C and 60% relative humidity. Tobacco growth was measured under light-emitting diodes (LEDs) (100 μmol m^−2^ s^−2^) as the light source. arabidopsis RNAi transgenic plants suppressing both *MPK3* and *MPK6* genes (*mpk3/6*) have been described previously [28]. We obtained the *rbohD/F* double-knockout arabidopsis mutant from Dr. Jonathan Jones (Norwich, UK).

### 2.3. Nitrotetrazolium Blue (NBT) Staining for Superoxide Determination

Superoxide (O_2_^●−^) was visualized by in situ histochemical staining using NBT. *Arabidopsis thaliana* leaves were collected at 30 min post-infiltration with melatonin or 2-OHM, followed by immersion in a solution containing 0.1% NBT (10 mM MES, pH 6.8) for 4 h. The stained leaves were decolorized and stored in 96% ethanol.

### 2.4. Transient Expression of OsM2H in Tobacco Leaves

The pER-mCherry and pBIN61-GFP-HA (P35s:GFP-HA) vectors, kindly donated by Dr. H.G. Kang (Texas State University, San Marcos, TX, USA), were used for estradiol-inducible transient expression of OsM2H:mCherry fusion protein in tobacco (*N. benthamiana*) leaves. The pER-OsM2H:mCherry vector construction was previously described in [29]. *Agrobacterium tumefaciens* strain GV2260 harboring either the pER-OsM2H:mCherry or pBIN61-GFP-HA plasmid was infiltrated into tobacco leaves. Infected leaves were treated with estradiol (10 µM) 2 days after infiltration, followed by 12 h of incubation for the NBT assay. β-estradiol was obtained from Sigma-Aldrich (St. Louis, MO, USA).

### 2.5. Melatonin and 2-OHM Treatment

Melatonin and 2-OHM were purchased from Sigma-Aldrich and Toronto Research Chemicals (North York, ON, Canada), respectively. Stock solutions (1 mM) of melatonin or 2-OHM were dissolved in 2 mM MES buffer (pH 5.6 in 5 mM MgCl_2_) and diluted with the same buffer used for infiltration. The first or second leaves from 6-week-old arabidopsis plants grown under standard conditions (50 μmol m^−2^ s^−1^) were infiltrated with melatonin or 2-OHM (5 mM MgCl_2_ in 2 mM MES, pH 5.6) using a needleless 1-mL syringe and transferred to either dark or low light conditions (7 μmol m^−2^ s^−1^), followed by sample harvesting at various time intervals. For the ion leakage analysis, melatonin and 2-OHM were infiltrated into the abaxial sides of arabidopsis leaves. Plants challenged with either melatonin or 2-OHM were immersed in distilled H_2_O for 5 min to eliminate signals derived from wounded cells. Then, the samples were placed in 50-mL tubes containing 15 mL of distilled H_2_O and solution conductivity was measured using a conductivity meter (Cole-Parmer Instrument Co., Vernon Hills, IL, USA).

### 2.6. Protein Extraction and Protein Gel Blotting Analysis

The first and second arabidopsis leaves were sampled at 48 or 72 h post-infiltration with 2-OHM (10 µM) at 24 h for protein analysis. Protein extracts were prepared with 40 mM HEPES, pH 7.5, 100 mM NaCl, 1 mM EDTA, 10% glycerol, 0.2% Triton X-100, and 1 × Roche Protease Inhibitor Cocktail (Roche Applied Science, Indianapolis, IN, USA), and then centrifuged at 10,000× *g* for 10 min at 4 °C. Aliquots of the supernatant were mixed with sample buffer (Tris-HCL, pH 6.8, 10% sodium dodecyl sulfate [SDS], 10 mM DTT, 20% glycerol, and 0.05% bromophenol blue). SDS-polyacrylamide gel electrophoresis (PAGE) and a blot assay were performed as described previously in [27]. Antibodies against Lhcb1, Lhcb4, RBCL, and ClpR1 were purchased from Agrisera (Vannas, Sweden).

### 2.7. RNA Analysis

Total RNA was extracted from leaves using a Nucleospin RNA Plant Kit (Macherey-Nagel, Duren, Germany). Reverse transcription was performed using a Stratagene Reverse Transcription Kit (Stratagene, La Jolla, CA, USA). Real-time PCR (qRT-PCR) was performed using a Mic qPCR Cycler System (Bio Molecular Systems, Queensland, Australia) using a SYBR Green RT-PCR Reagent Kit (Luna Universal qPCR Master Mix; NEB, Hitchin, UK) according to the manufacturer’s protocol. We used *Cyclophilin* (*Cyclo*) or *Nt16s* rRNA as a normalization control for qRT-PCR and reverse-transcription (RT)-PCR. The RT-PCR conditions were as described previously in [30]. The primer sequences for RNA expression analysis are listed in Table S1.

### 2.8. High-Performance Liquid Chromatography (HPLC) Analysis for 2-OHM Measurements

Tobacco leaves (100 mg) were ground to a powder in liquid nitrogen using a Tissuelyser II system (Qiagen, Tokyo, Japan) and extracted with 1 mL of chloroform. The chloroform extracts were evaporated until dry and dissolved in 200 µL of 40% MeOH. Aliquots of 20 µL were subjected to HPLC with an ultraviolet (UV) detector system (Waters, Milford, MA, USA) as described previously in [31]. Briefly, the samples were separated using a Sunfire C18 column (Waters; 4.6 × 150 mm) using isocratic elution with 15% MeOH in 0.3% trifluoroacetic acid at a flow rate of 1 mL/min. We detected 2-OHM at 254 nm. All measurements were conducted in triplicate.

### 2.9. Statistical Analyses

Means were compared using analysis of variance (ANOVA) with IBM SPSS Statistics 25 software (IBM Corp. Armonk, NY, USA). Means with significant differences were identified using a post-hoc Tukey’s honest significant difference (HSD), at a level of *p* < 0.05. Data are presented as means ± standard deviation (SD).

## 3. Results

### 3.1. Generation and Characterization of Transgenic Tobacco Overexpressing Rice M2H

**Figure 1:**
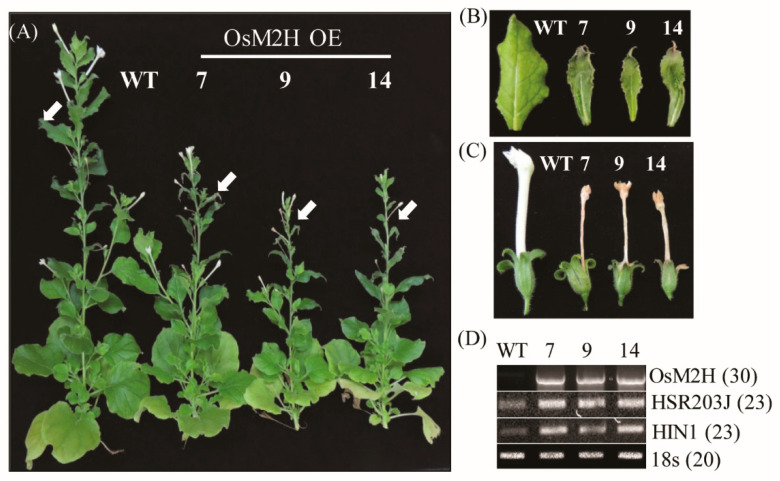
Overexpression of *OsM2H* caused dwarfism and spontaneous cell death in transgenic tobacco (*Nicotiana benthamiana*). (**A**) Phenotypes of *OsM2H* overexpression (OE) in tobacco. Tobacco plants grown in soil under long-day growth conditions (16 h light/8 h dark) at 28 °C for up to 12 weeks were photographed. (**B)** Phenotypes of upper tobacco leaves, indicated by arrows in (**A**). (**C**) Flower phenotypes. (**D**) Reverse-transcription polymerase chain reaction (RT-PCR) analysis results. The GenBank accession numbers of *OsM2H, HSR203J*, and *HIN1* are AK119413, AB091430, and Y07563, respectively. Numbers in parentheses indicate the number of PCR cycles.

Previously, we attempted to generate transgenic rice overexpressing the rice *M2H* gene (*OsM2H*) but failed because embryogenic transgenic calli were necrotized during the regeneration process, leading to lethality during somatic embryogenesis [24]. In this study, we attempted to generate *M2H* overexpression plants through organogenesis regeneration using a tobacco transformation system. We successfully generated transgenic tobacco plants without the hindrance of transgenic tobacco callus organogenesis. From 14 independent T_1_ transgenic tobacco plants, we further selected three homozygous tobacco plants. These T_2_ tobacco transgenic plants were grown to maturity (12 weeks) and showed a retarded growth phenotype compared to the wild type (WT) (Figure 1A). The upper leaves of transgenic tobacco also showed necrotized and cell-death phenotypes in conjunction with senesced flowers compared with the WT (Figure 1B,C). Notably, the flowers of the transgenic tobacco were smaller, with shorter corolla tubes than those of WT, but their necrotic corollae were still attached to the receptacle until the later stages of flower development. Several cell death marker genes, such as hypersensitivity-related gene (*HSR203J*) and harpin-induced 1 (*HIN1*) [32], were dramatically induced in these transgenic upper leaves compared to those of the WT (Figure 1D). The lower leaves of tobacco showed more advanced senescence in the transgenic plants than in the WT. Thus, *M2H* overexpression clearly resulted in premature senescence or leaf necrosis.

**Figure 2:**
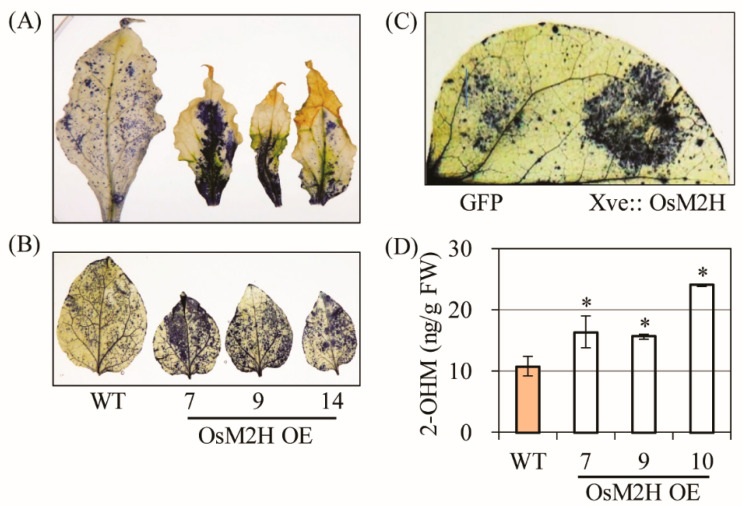
(**A**) Determination of superoxide levels by nitrotetrazolium blue (NBT) staining in upper leaves from 12-week-old transgenic tobacco plants. (**B**) Determination of superoxide levels by NBT staining in 6-week-old lower transgenic tobacco leaves. (**C**) Determination of superoxide levels by infiltrating *Agrobacterium* strains harboring the XVE-inducible OsM2H-Cherry or 35 s-GFP-HA plasmids. Six-week-old wild-type (WT) tobacco leaves were induced by estradiol (10 µM) treatment, followed by 10 h of incubation prior to NBT staining. (**D**) High-performance liquid chromatography (HPLC) quantification of 2-OHM in upper leaves of tobacco (Figure 1B). Error bars indicate the standard deviation of three biological replicates. Asterisks (*) indicate significant differences from the WT control, determined using Tukey’s post-hoc honest significant difference (HSD) test at a level of *p* < 0.05.

All necrotic leaves had greatly enhanced superoxide levels compared with the WT according to our NBT staining results (Figure 2A), and young (6 weeks) tobacco leaves not showing senescence symptoms also exhibited higher superoxide levels in *OsM2H* transgenic tobacco than in the WT (Figure 2B). To determine whether ROS production was directly coupled with the *OsM2H* gene in these plants, we infiltrated *Agrobacterium* strains harboring *OsM2H* under the control of the estrogen-inducible XVE promoter. Upon β-estradiol induction for 12 h, transient *OsM2H* expression led to an increase in ROS production compared to the control green fluorescent protein (*GFP*) gene, suggesting that ROS production occurred not only in transgenic tobacco expressing *M2H* constitutively, but also in tobacco leaves expressing *M2H* transiently (Figure 2C). Consistent with the close relationship between *M2H* and ROS production, 2-OHM content was higher in the *OsM2H* transgenic tobacco leaves than in the corresponding WT (Figure 2D). Together, these data clearly suggest that 2-OHM, the enzymatic product of the M2H enzyme, plays a direct role in ROS production and is responsible for the premature senescence of *OsM2H* transgenic tobacco plants.

### 3.2. Production of Superoxide upon 2-OHM Treatment in Arabidopsis Leaves

**Figure 3:**
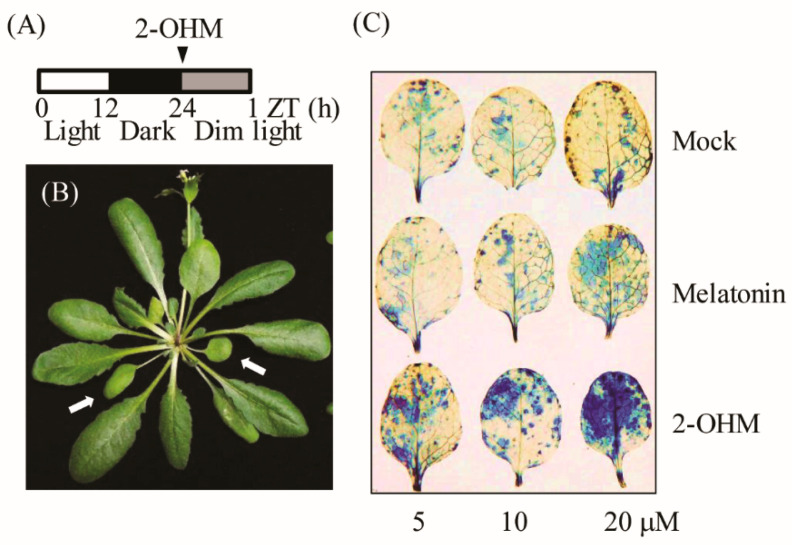
(**A**) Schematic of 2-OHM treatment in arabidopsis leaves under dim light conditions (7 µmol m^−2^ s^−1^). (**B**) Arabidopsis leaves (arrows) grown for 6 weeks (first or second leaves) were used for 2-OHM treatment. (**C**) Determination of superoxide levels by NBT staining in response to varying levels of 2-OHM and melatonin. Mock treatment consisted of 5 mM MgCl_2_ in MES (2 mM, pH 5.6).

To elucidate the direct relationship between 2-OHM and ROS, varying concentrations of 2-OHM or melatonin were independently infiltrated into first and second arabidopsis leaves and incubated for 60 min under dim light conditions (7 μmol m^−2^ s^−1^). Superoxide levels were visualized by NBT staining. Dense NBT staining was observed in arabidopsis leaves treated with 2-OHM in a dose-dependent manner (Figure 3), whereas mock treatments showed no visible staining (Figure 3C). Melatonin treatment at a concentration of 20 μM resulted in slight staining. These data clearly demonstrate that ROS was mainly produced by 2-OHM, rather than melatonin, indicating that 2-OHM is the key molecule involved in ROS production. The low ROS production in response to melatonin treatment may be attributed to the conversion of melatonin into 2-OHM in arabidopsis leaves. ROS production in response to 2-OHM was barely observed in either young or rapidly growing arabidopsis leaves or old arabidopsis leaves under normal light conditions (50 μmol m^−2^ s^−1^), suggesting that 2-OHM is involved in senescence-related ROS production. These findings are consistent with previous reports that senescence is age-dependent and inhibited by light [33,34,35].

### 3.3. Differences in Gene Expression Patterns Elicited by Exogenous 2-OHM Treatment and Exogenous Melatonin Treatment

**Figure 4:**
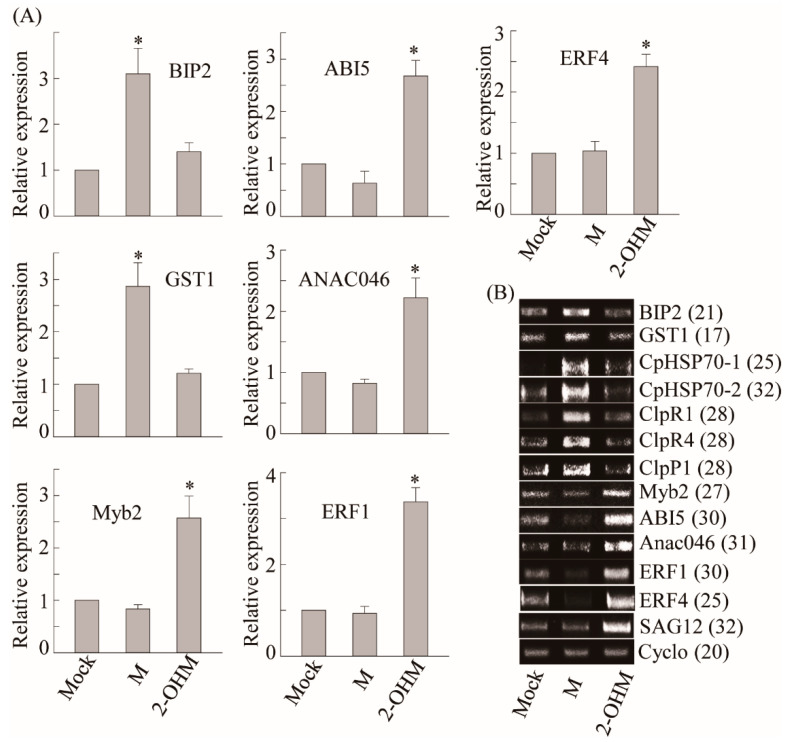
(**A**) Real-time PCR (qRT-PCR) analysis results for various genes involved in cell homeostasis and ABA and ET signaling in arabidopsis leaves in response to melatonin or 2-OHM treatment. (**B**) RT-PCR analysis results. Some genes analyzed by qRT-PCR were further analyzed by RT-PCR for clarification. Six-week-old Col-0 WT arabidopsis leaves (first or second leaves) were infiltrated with either melatonin (M; 1 μM) or 2-OHM (1 μM) and then incubated under dim light conditions (7 µmol m^−2^ s^−1^) for 2 h before leaves were collected. Mock treatment consisted of 5 mM MgCl_2_ (2 mM MES, pH 5.6). Expression levels were normalized to *Cyclo* for qRT-PCR. Asterisks indicate significant differences from the mock control as determined by Tukey’s post-hoc HSD test at a level of *p* < 0.05. GenBank accession numbers were as follows: *BIP2* (AT5g42020), *GST1* (AT1g02920), *CpHSP70.1* (AT4g24280), *CpHSP70.2* (AT5g49910), *ClpR1* (AT1g49970), *ClpR4* (AT4g17040), *ClpP1* (ATCg00670), *Myb2* (AT2g47190), *ABI5* (AT2g36270), *Anac046* (AT3g04060), *ERF1* (AT3g23240), *ERF4* (AT3g15210), *SAG12* (AT5g45890), and *Cyclo* (AT4g38740). Numbers in parentheses indicate the number of PCR cycles.

To compare differential gene expression patterns between plants treated with melatonin and 2-OHM, we selected a series of melatonin-induced genes including ROS defense-related genes such as *GST1* and protein homeostasis-related genes such as heat shock protein (*CpHSP70*) and caseinolytic protease (*Clp*) [27]. Exogenous melatonin treatment (1 μM) induced a number of genes including *GST1, BIP2, CpHSP70-1, CpHSP70-2, ClpR1, ClpR4,* and *ClpP1*, as described previously (Figure 4) [27]. However, no genes induced by melatonin treatment were induced by 2-OHM treatment (1 μM), indicating the distinctive signaling roles of 2-OHM and melatonin. Based on ROS generation by 2-OHM, we monitored an array of genes involved in cell death and senescence that are associated with ethylene and ABA. The mRNA expression levels of ABA-insensitive 5 (*ABI5*), MYB domain protein 2 (*Myb2*), and NAC domain-containing protein 46 (*ANAC046*), which are major transcription factors involved in ABA signaling, were greatly enhanced in response to 2-OHM. In contrast, these genes were downregulated by melatonin treatment. Two ethylene response transcription factors, *ERF1* and *ERF4*, showed higher mRNA levels following 2-OHM treatment, but were suppressed by melatonin treatment. The expression levels of senescence-associated gene 12 (*SAG12*), which is a representative senescence marker gene, were also greatly induced by 2-OHM, whereas no such induction was observed in response to melatonin. Based on these findings, 2-OHM is clearly a positive factor in senescence, whereas melatonin is a closely associated negative factor in senescence [27]. Although 2-OHM is a simple melatonin derivative, it played completely different signaling roles than melatonin.

### 3.4. Ethylene and ABA Signaling Was Required for 2-OHM-Induced Gene Expression

**Figure 5:**
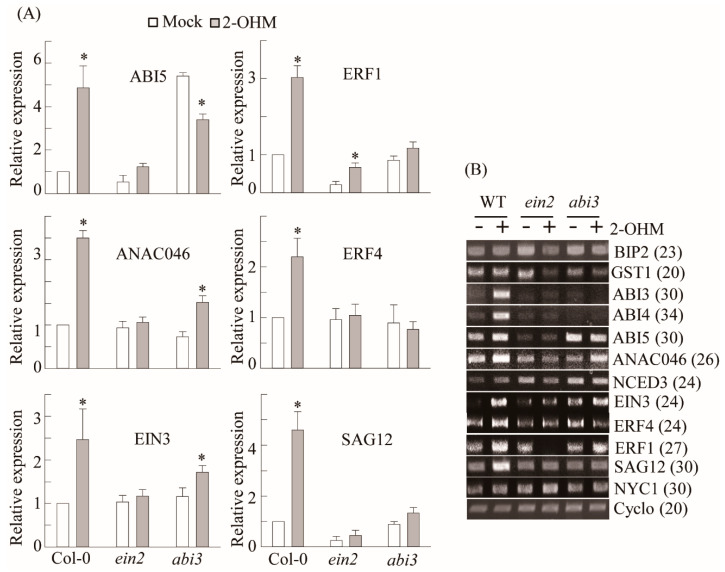
EIN2- and ABI3-dependent gene induction in response to 2-OHM treatment. (**A**) qRT-PCR analysis results. (**B**) RT-PCR analysis results. Arabidopsis WT (Col-0), *ein2*, and *abi3* plants were treated with 2-OHM (1 µM). Leaves were infiltrated with solutions and incubated under dim light conditions for 2 h prior to sample harvesting. Mock treatment consisted of 5 mM MgCl_2_ in 2 mM MES (pH 5.6). Cyclo was used for qRT-PCR normalization and as a loading control for RT-PCR. GenBank accession numbers were as follows: *ABI3* (AT3g24650), *ABI4* (AT2g40220), *NCED3* (AT3g14440), *EIN3* (AT3g20770), and *NYC1* (AT4g13250). Other genes are listed in Figure 4. Asterisks (*) indicate significant differences from the mock control, determined using Tukey’s post-hoc HSD test at a level of *p* < 0.05. Numbers in parentheses indicate the number of PCR cycles.

To determine whether 2-OHM-mediated gene induction of ethylene and ABA-related transcription factors is dependent on ethylene and ABA signaling, we employed knockout mutant lines of *EIN2* and *ABI3*, which are key signaling factors of ethylene and ABA [36]. Ethylene-related transcription factors including *EIN3*, *ERF1*, and *ERF4*, which were induced by 2-OHM treatment, were abolished in the *ein2* mutant (Figure 5). Similarly, ABA-related transcription factors such as *ABI3*, *ABI4*, and *ABI5* failed to be induced by 2-OHM in the *abi3* mutant and *NCED3*, an ABA biosynthetic gene that encodes 9-cis-epoxycarotenoid dioxygenase 3, was slightly increased upon 2-OHM treatment, whereas this induction was reversed slightly in the *abi3* mutant. The induction of *ANAC046*, an NAC transcription factor and positive regulator of chlorophyll degradation, was abolished in the *ein3* mutant, whereas its expression was significantly reduced in the *abi3* mutant. In contrast, NON-YELLOW COLORING 1 (*NYC1*), which is involved in chlorophyll degradation, was not responsive to 2-OHM treatment. These data suggest that 2-OHM plays more important roles in ethylene and ABA signaling cascades than in their biosynthetic pathways.

### 3.5. RBOH-Dependent ROS Production and Ethylene and ABA Signaling Requirement of 2-OHM

**Figure 6:**
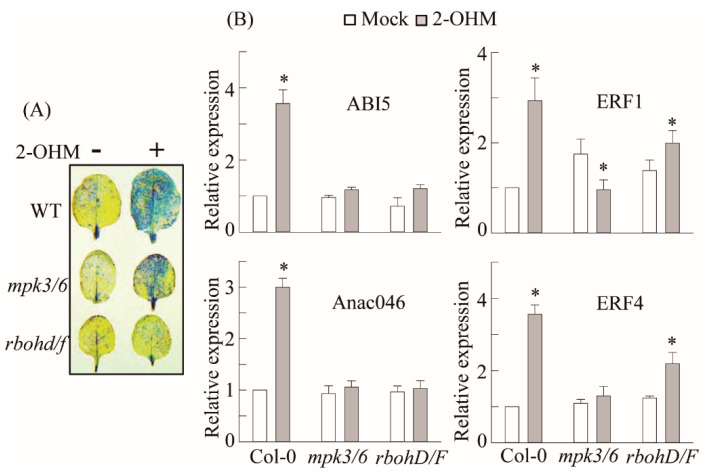
Involvement of MPK3/6 and RHOHD/F in 2-OHM-mediated superoxide and senescence-related gene induction. (**A**) Determination of superoxide levels by NBT staining. (**B**) qRT-PCR analysis results for ethylene and ABA signaling-related genes. Six-week-old WT, *mpk3/6*, and *rbohD/F* leaves were treated with 2-OHM (1 µM) as described for Figure 3. (*)Asterisks indicate significant differences from the mock control, as determined by Tukey’s post-hoc HSD test, at a level of *p* < 0.05.

In plants, ROS are generated by either RBOH located in the plasma membrane or photo-activated chloroplasts. Some studies have suggested that melatonin may induce RBOH in plants [19,21]. Many melatonin-mediated defense responses against pathogen [28], high-light [37], low-light [27], and ER [38] stress are mediated by the mitogen-activated protein kinase (MPK) pathway. Therefore, we examined the possible involvement of RBOH and MPK3/6 in 2-OHM-mediated ROS production as well as ethylene and ABA signaling in arabidopsis. First, we measured superoxide production following 2-OHM treatment in either the *rbohD/F* double knockout mutant or the *mpk3/6* double RNAi line. Superoxide production following 2-OHM treatment was completely arrested in the *rbohD/F* mutant, but not in the *mpk3/6* RNAi line, when compared to WT Col-0, suggesting the absolute dependency of 2-OHM-mediated ROS production on RBOH, but not MPK3/6 (Figure 6). Next, we monitored gene expression levels associated with ethylene and ABA signaling. All of these genes were barely induced in the *rhohD/F* and *mpk3/6* lines in response to 2-OHM treatment (Figure 6B), indicating the strong dependence of 2-OHM-mediated ethylene and the ABA signaling pathways on both RBOH and MPK3/6. Although MPK3/6 is not essential for ROS generation by 2-OHM, MPK3/6 is critical for the induction of ABA and ethylene-related transcription factors. These data indicate that both melatonin and 2-OHM accept the MPK pathway as an integrated mediator to activate their own distinctive signaling. These findings also suggest that the ROS RBOHD/F acts upstream of MPK3/6 signaling when arabidopsis leaves are exogenously treated with 2-OHM.

### 3.6. Acceleration of Dark-Induced Senescence upon 2-OHM Treatment in Arabidopsis

**Figure 7:**
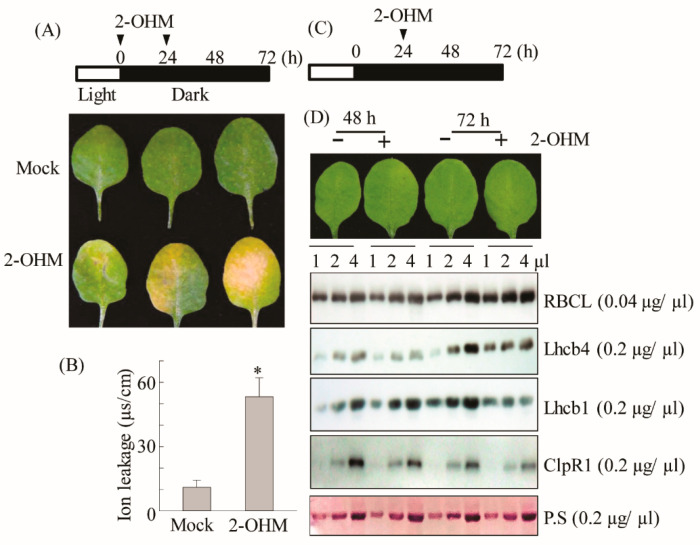
(**A**) Schematic diagram of 2-OHM treatment for senescence induction and the resulting phenotypes. (**B**) Ion leakage measurement. (**C**) Schematic diagram of 2-OHM treatment for physiological tests. (**D**) Western blot analysis using anti-RBCL, -Lhcb4, -Lhcb1, and -ClpR1 antibodies in the WT in response to 2-OHM treatment. We administered 2-OHM (10 µM) into Col-0 leaves twice (0 and 24 h) as described for Figure 3. Leaves were collected at 72 h for photography (**A**) and ion leakage measurements (**B**). A single treatment of 2-OHM was administered at 24 h to measure protein levels involved in chloroplast homeostasis. Total leaf protein extracts were subjected to 14% sodium dodecyl sulfate-polyacrylamide gel electrophoresis (SDS-PAGE). The immunoblot was probed with specific antibodies (right). Bottom panel shows a loading control stained with Ponceau S solution (P.S). Mock treatment consisted of 5 mM MgCl_2_ in 2 mM MES (pH 5.6). (*) Asterisks indicate significant differences from the mock control, determined by Tukey’s post-hoc HSD test at a level of *p* < 0.05.

Based on ROS production and ethylene and ABA signaling gene induction by 2-OHM, we hypothesized that 2-OHM could be a senescence-inducing factor. To test this hypothesis, the first or second arabidopsis leaves of 6-week-old plants were infiltrated abaxially with 10 μM 2-OHM twice (at 0 and 24 h), followed by incubation in the dark to monitor darkness-induced senescence symptoms. We found that 2-OHM triggered clear leaf chlorosis and increased ion leakage levels (Figure 7A,B); these symptoms were not observed under light or in young or mature arabidopsis leaves (data not shown), suggesting that 2-OHM does not act as a senescence-inducing signal under daylight conditions or in young leaves. Because M2H protein is localized in chloroplasts [29], 2-OHM is likely first produced within chloroplasts. To determine whether 2-OHM affects chloroplast function, we monitored the expression levels of the light-harvesting antenna protein Lhcb1 and the key chloroplast molecular chaperone ClpR1 (caseinolytic protease) in response to 2-OHM treatment under dark incubation. The Lhcb1 level affects chlorophyll levels and state transition in arabidopsis [39], and ClpR1 is essential for chloroplast maintenance by controlling Lhcb2 protein levels, such that the ClpR1-knockout mutant results in an abnormal arabidopsis phenotype [40]. We administered one treatment of 2-OHM (10 μM) at 24 h after a dark incubation period (Figure 7C) to avoid the severe senescence symptoms shown in Figure 7A. Leaves harvested at 48 and 72 h after dark incubation were assayed for protein levels. The protein levels of ribulose-1,5-bisphosphate carboxylase/oxygenase (RBC) large subunit (RBCL) were not affected in 2-OHM-treated leaves compared with the mock control leaves. However, the expression levels of Lhcb1, Lhcb4, and ClpR1 proteins were reduced in leaves treated with 2-OHM compared with those in the mock control. These results indicate that 2-OHM treatment induces protein instability of chloroplast maintenance components, eventually leading to rapid necrotic chlorosis under darkness-induced senescence. In marked contrast, the opposite results were achieved by melatonin treatment of arabidopsis leaves, which increased the expression levels of Lhcb1, Lhcb4, and ClpR1 proteins [27].

## 4. Discussion

Based on the hypothesis that the plant *SNAT* genes stem from cyanobacteria, which were the ancestors of chloroplasts, it has become widely accepted that all plants harboring chloroplasts have the capacity to synthesize melatonin [41]. Unlike early reports of high melatonin content in various plants [42], recent studies have shown that plants are able to synthesize very low levels of melatonin, ranging from pg/g fresh weight (FW) to a few ng/g FW, closely matched by the very low catalytic activity of plant SNAT enzymes, the penultimate enzymes for melatonin biosynthesis [8,43,44]. Although melatonin is produced in very low levels in plants, it plays a wide array of physiological roles through its potent antioxidant activity, and it acts as a signaling molecule responsible for the induction of a large number of genes involved in ROS detoxification [45], pathogen defense [46], abiotic stress tolerance [47], protein quality control [27], and growth modulation [48,49]. Although melatonin is commonly thought to be a growth stimulant and defense signaling molecule against adverse stimuli in plants, it plays a controversial role in ROS synthesis induction in plants [19,21], although its possible involvement as a pro-oxidant has been demonstrated in animals [23].

In this study, we report for the first time that 2-OHM, not melatonin, plays a pro-oxidant role in inducing ROS production upon exogenous treatment in arabidopsis (Figure 3), as well as in transgenic tobacco overproducing 2-OHM (Figure 1 and Figure 2). An in vitro study was the first to observe that 2-OHM acted as a melatonin oxidation product in the presence of hypochlorous acid [50]; a later study found the same effect in UV-induced skin cells [51]. In plants, 2-OHM was first found in rice roots treated with 1 mM melatonin after the successful cloning of *M2H* from rice [31], followed by a report that 2-OHM was produced in 24 plant species at levels 300-fold higher than melatonin on average, suggesting that 2-OHM is a major melatonin derivative in plants [12]. Due to its higher levels in plants, 2-OHM was initially thought to be simply a nonfunctional byproduct or inactive form of melatonin in plants. However, 2-OHM treatment induced plant defense genes, although to a smaller extent than melatonin, in arabidopsis [11] and conferred tolerance against combined cold and drought stress in several plants [52,53]. In cucumber plants, 2-OHM treatment ameliorated cadmium toxicity by enhancing antioxidant synthesis [54] and antioxidant enzymes [55]. To date, no studies have explored the possible roles of 2-OHM in ROS production or either ABA or ethylene signaling components in plants and animals. The first suggestion that 2-OHM produced ROS was observed indirectly in transgenic rice calli overexpressing the rice *M2H* gene, which exhibited cell death or necrosis during somatic embryogenesis, resulting in the failure to acquire transgenic rice plants [24]. Instead of plant regeneration via somatic embryogenesis as occurs in rice transformation, we attempted to overexpress the rice *M2H* gene via organogenesis, similar to tobacco transformation, and successfully generated transgenic tobacco plants overexpressing rice *M2H* (Figure 1 and Figure 2). These *M2H* transgenic tobacco plants exhibited early senescence and leaf necrosis symptoms indicative of ROS overproduction, suggesting the involvement of 2-OHM as a pro-oxidant.

**Figure 8:**
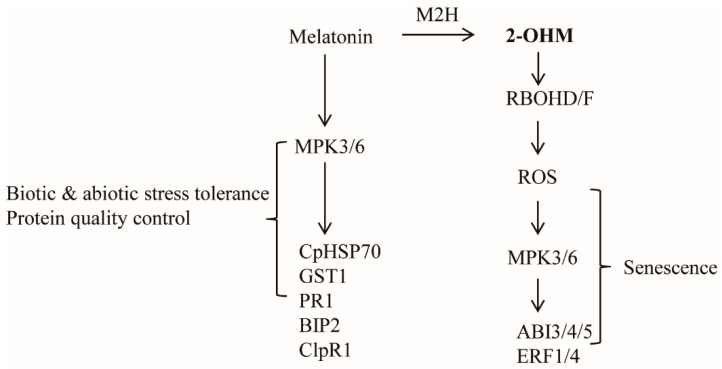
Proposed model of 2-OHM-mediated reactive oxygen species (ROS production and signaling pathway for senescence induction in arabidopsis. Melatonin is involved in defense responses against many biotic and abiotic stresses as well as in protein quality control, mediated by the MPK3/6 signaling pathway. Melatonin-induced genes included *CpHSP70*, *GST1, PR1, BIP2,* and *ClpR1*. Conversely, 2-OHM converted from melatonin by M2H induced ROS in an RBOHD/F-dependent manner, followed by the MPK3/6 signaling pathway, leading to the induction of many genes related to senescence signaling including *ABI3, ABI4, ABI5, ERF1,* and *ERF4*.

ROS are associated with senescence, which is subject to sophisticated genetic control in plants. Leaf senescence exhibits distinct tri-phase development in view of the key hormone ethylene, involving the no-senescence (early leaf growth), adaptive senescence, and always-senescence phases. Ethylene cannot induce leaf senescence during the early leaf stage, but promotes senescence during the adaptive phase, whereas senescence proceeds regardless of ethylene during the always-senescence phase [56]. In common with ethylene, 2-OHM does not induce senescence or ROS in the young mature leaves of arabidopsis, but promotes senescence and ROS in adaptive-phase leaves of arabidopsis (Figure 7), suggestive of a leaf age-dependent senescence activator of 2-OHM such as ethylene. In addition to ethylene alone, ABA also modulates leaf senescence in combination with ethylene, because ethylene signaling is involved in ABA-induced senescence [57,58]. Both ethylene and ABA also trigger ROS accumulation in plants, as does 2-OHM [59,60]. The precise balance between ROS production and scavenging in chloroplasts is important for photosynthesis and plant growth; otherwise, oxidative damage can lead to plant cell death or senescence [61]. Although the precise subcellular location of 2-OHM production remains to be investigated, it is likely that 2-OHM is predominantly produced in chloroplasts due to the chloroplast localization of corresponding M2H protein [11]. In this study, we found that, unlike melatonin, 2-OHM-induced ROS production in chloroplasts was absolutely dependent on RBOH, followed by the induction of a series of senescence signaling components such as ABIs and ERFs, leading to leaf senescence in arabidopsis. The induction of ethylene and ABA signaling components in response to 2-OHM is mediated by the MPK3/6 signaling pathway (Figure 6 and Figure 8), as melatonin-mediated pathogen defense requires the MPK3/6 pathway [28]. Interestingly, 2-OHM treatment in the adaptive leaf stage can induce leaf senescence by increasing protein instability, which is involved in chloroplast quality control, whereas melatonin has the opposite effect (Figure 7). Together, these results demonstrate for the first time that 2-OHM, not melatonin, is responsible for ROS production. The possible involvement of 2-OHM as a ROS producer in animal cells has also been addressed indirectly [62]. Further in-depth studies of enzyme activity, tissue distribution of 2-OHM, *M2H* mRNA expression profiles during plant growth and development, and responses to various stress conditions will reveal the key functions of 2-OHM in the context of ROS production in plants.

## 5. Conclusions

Melatonin is a potent antioxidant, and its application to plants tissues decreases ROS levels, leading to enhanced tolerance to many adverse stresses. In contrast, some studies have suggested that melatonin acts as a pro-oxidant, resulting in increased ROS production upon melatonin application to plants. In this study, we explored the potential role of melatonin as a pro-oxidant, in comparison with 2-OHM, a major melatonin metabolite synthesized by M2H. Our results, demonstrated that 2-OHM treatment induced ROS production, whereas melatonin treatment did not. Rice *M2H*-overexpressing transgenic tobacco plants exhibited dwarfism and necrosis in upper leaves and early senescence in lower leaves. We also showed that 2-OHM-mediated ROS production is RBOH-dependent and that 2-OHM-induced senescence genes require the ethylene and abscisic acid (ABA) signaling pathways in arabidopsis. In contrast to melatonin, 2-OHM treatment induced senescence symptoms such as leaf chlorosis and increased ion leakage in arabidopsis, accompanied by decreased levels of proteins involved in chloroplast maintenance including Lhcb1 and ClpR1. These results demonstrate that 2-OHM acts as a senescence-inducing factor by inducing ROS production in plants.

## Data Availability

The data presented in this study are available within the article.

## Supplementary Materials

The following are available online at https://www.mdpi.com/article/10.3390/antiox10111728/s1, Table S1: Sequences of primers in RNA analysis.
