# Supplementary material for: 2-Hydroxymelatonin, Rather Than Melatonin, Is Responsible for RBOH-Dependent Reactive Oxygen Species Production Leading to Premature Senescence in Plants
Source: Antioxidants (Basel). 2021 Oct 29;10(11):1728. doi: 10.3390/antiox10111728 (PMC8614918; doi:10.3390/antiox10111728)
Supplement: Supplementary file 1 [file antioxidants-10-01728-s001.zip › antioxidants-1449403-supplementary.pdf]

Supplemental Table S1. Sequences of primers in RNA analysis.

Gene	Forward primer (5′-3′)	Reverse primer (5′-3′)
BIP2	GCAGGAGGAGAATCATCGAC	AAAGAGAACGTCCAGGGAGA
GST1	GGACTCACCAAGCCTGTGTT	TGAATCGCATGAGTTTGACC
CpHSP70-1	GGAAGTGGATCAACCCAAGA	GCATCGATCACATCATCACC
CpHSP70-2	TTCGTCTTCTTCCTCCTCCA	GTATCGGGAAGCGTTGTTGT
ClpR1	CAATCCCTAATTTCGCCTCA	TGGAACAGACAGCTTCATGG
ClpR4	TCAGCGATTATCCCGTCTTC	CGTTTAGGCTTGGAACTGGA
ClpP1	TTCGAAGTCCTGGAGAAGGA	TTACCCATCCACCAGGAGAG
MYB2	AAACGGGTTTCGGAGAGTTC	TACACCAAACGTGACGCAAT
ABI3	TTTGGATCCTCCTTCGTCTC	ACCCAACTTGGCTATCATCG
ABI4	CGATGAGCATCCCTAAGGTC	TCTCCTCCGATTCTCTTCCA
ABI5	CCTCGTTCTGTCTCCGACTT	TGGAGAGGAAGAGGAAGCAA
NCED3	TACCAGTAAAATGCTCTGC	AGCTACAAGCTGGTCCCTCA
ANAC046	TATTCTTCAGCACCAACGTG	CAAATCCACCACCTCCTGAT
ERF1	GAGGAGCTTTTGGGGTCTTC	CAAACTGTTTTCGATGTTTCTCA
ERF4	CGCATGCGTTTCTTCTTCTT	ACATGGGGTGAAACCAAGTC
EIN3	CCTTCTTTTTCCCATCACCA	CAACAATGGAATCAGGCTCA
SAG12	GGAGGTGGTTTTGATTTCCA	GACATCAATCCCACACAAACA
NYC1	GTTAACAGACGCGATGGAGA	GCCTGGAAAAGAGCTAGGTG
OsM2H	ACTAGTATGCCCGCCGTGGCCGG	GGTACCTCAGGGTTTGTCGAG
NtHSR203J	AGCTATGAAAAAGGGGGAAA	AACCATTAGAACGTGACAATC
NtHIN1	GAGCCATGCCGGAATCCAAT	ACCAATCAATATGGCATCTGG
Nt16s	CGGCTACCACATCCAAGGAAGG	GAGCTGGAATTACCGCGGCTG
Cyclophilin	GTTCTTCATCTGCACCGTGA	AGACACCACAATGACGACGA
